# OA11 Treatment challenges and co-morbidity issues in a patient with Arthritis Mutilans

**DOI:** 10.1093/rap/rkac066.011

**Published:** 2022-09-28

**Authors:** Chooi Shawn Loh, Rizwan Rajak

**Affiliations:** Croydon Health Services NHS Trust, London, United Kingdom; Croydon Health Services NHS Trust, London, United Kingdom

## Abstract

**Introduction/Background:**

Psoriatic arthritis mutilans is known to be the most severe form of psoriatic arthritis^1^ and reported to have a prevalence of 2-21%^2^, depending on the classification used. Clinical features include shortening of the digits, telescoping and flail joints with radiographs showing severe osteolysis and bone resorption^3^. There is an increased prevalence of metabolic syndrome, cardiovascular disease and psychiatric illness among these patients^4^. Treatment of the disease is usually a stepwise approach starting with conventional synthetic disease modifying anti-rheumatic drug (csDMARD), combination csDMARD, biologic DMARD (bDMARD) or targeted synthetic DMARD (tsDMARD).

**Description/Method:**

This 65-year-old gentleman has a diagnosis of psoriatic arthritis, mutilans type for more than 40 years. His other current co-morbidities include hypertension, hyperlipidaemia and depression. He was treated with methotrexate with good efficacy until this was stopped in 2012. Whilst on methotrexate, he had deranged liver function tests and the ultrasound scan of his abdomen showed fatty infiltration of the liver. Liver biopsy showed steato-hepatitis which was thought to be secondary to methotrexate and the drug was stopped. He was then started on Sulphasalazine, which was uptitrated to 1.5 g twice a day but with no benefit. He was having 12 swollen and 8 tender joints with a patient global score of 6/10 and physician global score of 7/10.

The patient was started on Golimumab in August 2013. He had four doses of 50 mg and two doses of 100 mg of the drug. Each dose had resulted in severe headaches which necessitated discontinuation of the treatment. He was then commenced on Adalimumab in August 2014, which also resulted in severe headaches and sweating with no efficacy otherwise. At that point, his Alanine Transaminase (ALT) levels continued to be fluctuating between 50 to 80 U/L and he was also noted to be hypertensive.

The patient was then started on Ustekinumab in October 2016 to which he tolerated well and had almost immediate noticeable improvement in his tender and swollen joints. The patient remained stable on this three monthly treatment till this day.

Interestingly, the erosive changes on the x-rays of his hands and feet has not progressed since 2010, despite tolerance and efficacy challenges of his treatment.

Despite stability of his disease and treatment, this gentleman sadly attempted suicide in March 2021 by consuming weed killer. He was informally admitted to a mental health ward.

**Discussion/Results:**

We present a case of psoriatic arthritis mutilans which was complicated by side effects and lack of efficacy of several disease modifying treatment. He had methotrexate which had to be stopped due to steato-hepatitis. Both Golimumab and Adalimumab was stopped due to side effects characterised by headaches. Ustekinumab was started four years following the cessation of methotrexate which continues to be the most effective medication for this patient.

This patient also suffers with cardiovascular co-morbidities and depression. Tumour necrosis factor (TNF)-α associated endothelial dysfunction as well as interleukin (IL)-17A associated increased expression of adhesion and pro-inflammatory molecules may contribute to the association of psoriatic arthritis mutilans with cardiovascular disease^5,6^. It is also known that there is a higher prevalence of mental health disease in patients with psoriasis. This is thought to be due to the involvement of the same inflammatory pathway as proinflammatory IL-1 and IL-6 are increased in both psoriatic arthropathy and depression^7^.

**Key learning points/Conclusion:**

Based on our research, there is a lack of evidence for treatment specific to arthritis mutilans, due to the relatively rare nature of the disease. However, this case report has proved that treatment should be individualised to the patient, based on their response and tolerance to the medications.

It is also pertinent to be aware and manage co-morbidities associated with the disease which includes hypertension, hyperlipidaemia, diabetes mellitus and psychiatric illnesses. This requires close collaboration with the patient’s general practitioner.
